# Blockade of Treg derived TGF-β abrogates suppression of effector T cell function within the tumor microenvironment

**DOI:** 10.1186/2051-1426-1-S1-P173

**Published:** 2013-11-07

**Authors:** Sadna Budhu, David Schaer, Yongbiao Li, Alan Houghton, Samuel Silverstein, Taha Merghoub, Jedd Wolchok

**Affiliations:** 1 Immunology, MSKCC, New York, NY, USA; 2 Medicine, MSKCC, New York, NY, USA; 3 Columbia University, New York, NY, USA

## 

Regulatory T cells (Treg) play a role in suppression of anti-melanoma immunity; however, the exact mechanism is poorly understood. Through intravital two photon microscopy, we found that Pmel-1 effectors engage in cell-cell interactions with tumor resident Tregs. To determine if contact between Tregs and T effectors (Teff) hinders killing of tumor cells in vivo, we utilized ex-vivo three-dimensional collagen-fibrin gel cultures of B16 melanoma cells. Collagen-fibrin gel cultures recapitulated the in vivo suppression, rendering the dissociated tumor resistant to killing by in vitro activated antigen specific Teff. In vivo depletion of Tregs in foxp3-DTR mice prior to tumor excision reversed the suppression. Additionally, In vivo modulation of intra-tumor Tregs suppressive function by GITR ligation had a similar effect, leading to ex-vivo tumor killing. Using neutralizing antibodies, we found that blocking TGF-β reversed the suppression. In addition, soluble factors from collagen-fibrin gel tumors do not inhibit killing suggesting that suppression is contact or proximity dependent. The CD8 Teff recovered from these gels exhibit a decrease in Granzyme B expression and an increase in expression of T cell exhaustion marker PD-1. These findings support the conclusion that intra-tumor contact with Tregs during the effector phase of the immune response is responsible for inhibiting anti-melanoma immunity in a TGF-β dependent manner, elucidating a novel way to target intratumoral Tregs.

